# Long term all-cause mortality after myocardial infarction with non-obstructed vs obstructed coronary artery disease: a meta-analysis of adjusted data

**DOI:** 10.1186/s12872-023-03674-1

**Published:** 2024-01-02

**Authors:** Xueli Lu, Shengnan Zhu, Yanjiao Lu, Yanming Li

**Affiliations:** Cardiovascular Medicine, Huaihe Hospital of Henan University, Baogong Hubei Road, Kaifeng City, Henan Province China

**Keywords:** Myocardial infarction, Coronary artery disease, Mortality, MINOCA

## Abstract

**Background:**

The difference in the long-term outcomes of myocardial infarction in patients with non-obstructed coronary arteries (MINOCA) and patients with myocardial infarction with obstructed coronary artery disease (MI-CAD) is not clear. The current study aimed to pool adjusted data to compare long-term outcomes of MINOCA vs MI-CAD.

**Methods:**

Electronic literature search of PubMed, Embase, CENTRAL, and Google Scholar databases was done for publications up to 18th June 2023. Only studies reporting multivariable-adjusted data with > 1 year of follow-up were included.

**Results:**

Sixteen studies met the inclusion criteria. Our meta-analysis revealed no statistically significant difference in the risk of all-cause mortality between MINOCA and MI-CAD patients (HR: 0.90 95% CI 0.68, 1.19 I^2^ = 94% *p *= 0.48). Analysis of the limited data showed a reduced combined risk of all-cause mortality and MI (HR: 0.54 95% CI 0.39, 0.76 I^2^ = 72% *p* = 0.003) and major adverse cardiac events (MACE) (HR: 0.66 95% CI 0.51, 0.84 I^2^ = 51% *p* = 0.0009) in patients with MINOCA vs MI-CAD, and no difference in the risk of cardiovascular mortality (HR: 0.81 95% CI 0.54, 1.22 I^2^ = 0% *p* = 0.31) and readmission between the two groups (HR: 0.85 95% CI 0.61, 1.19 I^2^ = 90% *p* = 0.35).

**Conclusion:**

A pooled analysis of adjusted outcomes from the available studies indicated that MINOCA and MI-CAD patients have similar long-term all-cause mortality risk. Our conclusions on the risk of cardiovascular mortality, MACE and readmission rates need to be taken with caution due to a lack of adequate studies. Further research is needed to strengthen the evidence on this important subject.

**Supplementary Information:**

The online version contains supplementary material available at 10.1186/s12872-023-03674-1.

## Introduction

Acute myocardial infarction (MI) accounts for a significant portion of morbidity and mortality cases around the world [1]. Studies indicate that compared to general population, patients with MI are at 30%-higher risk of mortality and adverse cardiovascular events [2]. The use of coronary angiography during the early management of this disease significantly improves identification of patients with MI and non-obstructed coronary arteries (MINOCA) [3]. A systematic review by Pasupathy et al. [4] indicated that the prevalence of MINOCA is around 6%, ranging between 1 and 14%. Patients with MINOCA tend to be younger, of the female sex, and with lower incidence of hyperlipidaemia compared to patients with MI and obstructed coronary artery disease (MI-CAD) [4].

Based on the guidelines of the European Society of Cardiology, diagnosis of MINOCA requires evidence of MI along with the demonstration of < 50% stenosis on a coronary angiogram [5, 6]. Management of MINOCA is challenging as the apparent reason of MI is not very clear. The disease is heterogeneous without any single pathophysiological mechanism [3]. Studies have reported that factors such as vasospasm of coronary vasculature, thrombosis or embolism, microvascular dysfunction, plaque disturbance, and supply–demand inadequacy may all lead to MI in these patients [7, 8]. Due to the unique nature of the disease and the difference in the mechanism of myocardial injury it is imperative to understand if the prognosis of MINOCA patients differs from that of MI-CAD.

Recently, several publications have compared outcomes of MINOCA and MI-CAD but with variable results. Some authors have reported lower mortality rates in patients with MINOCA [9–11], while others indicate no difference in outcomes between the two [12, 13]. A meta-analysis by Pelliccia et al. [14] have attempted to compare mortality rates between the two conditions. However, a significant drawback of this review is that only crude death rates were pooled. The observed difference in outcomes between MINOCA and MI-CAD may be, therefore, due to the difference in clinicopathological features of the diseases or, alternatively, because of the difference in several other risk factors [12]. Therefore, assessing the risk of mortality requires careful accounting for confounding factors. To date, no meta-analysis has compared outcomes of MINOCA and MI-CAD by aggregating multivariable-adjusted data. Current study aims to evaluate if there was a difference in long-term mortality between MINOCA vs MI-CAD by pooling only multi-variable adjusted data.

## Material and methods

This review conforms with the guidelines of the PRISMA statement [15]. Registration of protocol was done on PROSPERO (CRD42023436897). No ethical clearance or patient consent was required for this study.

### Search strategy

Two reviewers conducted a literature search for relevant studies in the PubMed, Embase, CENTRAL, and Google Scholar databases. It was completed on 18th June 2023. Keywords used were: “MINOCA”, “myocardial infarction”, “normal”, “non-obstructed”, “absence”, “obstruction”, “coronary artery”, and “coronary stenosis”. The combinations used are shown in Table 1. The retrieved studies were de-duplicated, and titles and abstracts were screened to remove non-relevant publications. Full-text analysis of the selected studies was done, and studies fulfilling all the criteria were included in the final analysis. All disputes were resolved by consultation. Hand search was also done for the bibliography of eligible studies.

**Table 1:** Search strategy

Search number	Query
1	(((normal) OR (non-obstructed)) AND (coronary stenosis)) AND (myocardial infarction)
2	(((absence) AND (obstruction)) AND (myocardial infarction)) AND (coronary artery)
3	(((normal) OR (non-obstructed)) AND (coronary artery)) AND (myocardial infarction)
4	(MINOCA)

### Inclusion criteria

The review question according to PICO was: Are the long-term *outcomes* of patients with MI (*population*) due to non-obstructed coronary artery disease (*intervention*) different as *compared* to those with obstructed coronary artery disease?

The inclusion criteria were then framed based on the above question as follows:

1) All kinds of studies comparing outcomes of MINOCA and MI-CAD. 2) Studies with a follow-up of at least 1 year. 3) Studies reporting multivariable-adjusted outcomes and specifying the factors adjusted for the analysis. 4) Studies were to diagnose MI based on typical symptoms, increase of a minimum of one necrosis biomarker, and ST-segment or T-wave changes on the electrocardiogram. 5) Patients were to be classified into MINOCA or MI-CAD groups based on the angiographic assessment of coronary arteries.

Studies excluded were: 1) Studies wherein angiographic assessment was not carried out. 2) Studies without adjusted outcomes. 3) Studies on Takotsubo syndrome 4) Non-English language studies. 5) Studies with duplicate or overlapping data. In such cases, the study with maximum patients was selected.

### Data management and quality assessment

Name of the author, study type, its location, number of patients, age, gender, medical history of patients (hypertension, diabetes mellitus, chronic kidney disease, previous MI or cerebrovascular accident), medications prescribed at discharge (aspirin, P2Y12 inhibitor, statins, beta-blockers), follow-up, and covariates examined were extracted from the included studies. The primary outcome was all-cause mortality after 1 year of follow-up. Secondary outcomes were cardiovascular mortality, the combined risk of mortality and MI, risk of major adverse cardiac events (MACE), and readmission rates between the two groups. MACE was defined as per the included studies. There was no restriction on the cause of readmission; all causes of readmissions were admissible.

Studies were examined for bias using the Newcastle–Ottawa scale (NOS) [16]. The scale judges each study for selection of study participants, comparison of study groups, and outcomes. The score of NOS ranges from 0–9.

### Statistical analysis

"Review Manager" (RevMan, version 5.3) was used for all quantitative data analyses. Adjusted hazard ratios (HR) and their 95% confidence intervals (CI) or similar effect sizes were combined by the generic inverse function of Review manager in a random-effects model. Publication bias was examined by visual inspection of funnel plots and Egger’s test. The I^2^ statistic determined the inter-study heterogeneity. A sensitivity analysis was executed by removing singular studies form the forest plot to check for any outliners. This was done in the software itself to note any change in significance of results.

## Results

### Search details

**Fig. 1:**
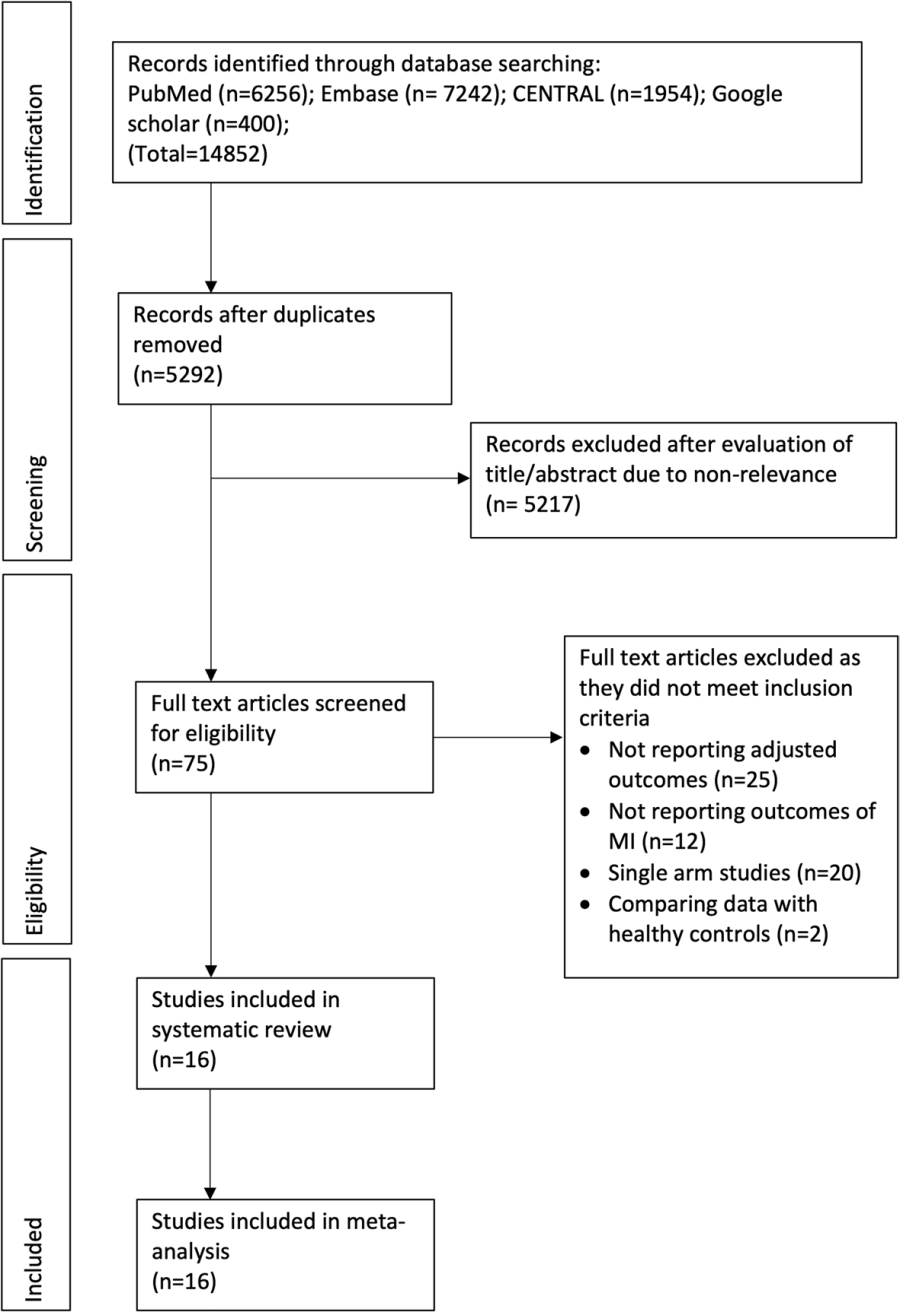
Study flow chart

Titles and abstracts of 5292 unique studies, identified by the search across the databases, were examined. Of them, 75 studies were selected for the full-text analysis. A total of 59 studies were excluded. Finally, 16 studies met the inclusion criteria [13, 17–31] (Fig. 1, Supplementary material: raw data).

### Study details

The studies were published between 2009 and 2023 (Table 2). Five studies were from the North America, two from Asia, two from New Zealand, and the remaining studies were from the European nations. The total number of patients in the MINOCA arm varied between 64 to 16,849. Sample sizes in the MI-CAD arm varied from 412 to 29,931. A total of 29,708 patients with MINOCA were compared with 514,421 patients with MI-CAD in the 16 studies. All studies were retrospective in design, examining data from hospital databases or national registries. Patients was mostly above 60 years old in most studies. Importantly, the study of Magnani et al. [24] had a younger cohort and the age of included patients was 38 and 41 years for MINOCA and MI-CAD groups, respectively. The percentage of hypertensive patients in the study groups ranged from 18.3 to 73.3%, while the percentage of diabetic patients varied from 3.8 to 37.9%. There was inconsistent reporting of data on previous MI and cerebrovascular accidents among the included studies. Medication-related data was also not provided by all included studies. However, a general trend noted was the reduced prescription of anti-platelets, statins, and beta-blockers at discharge in MINOCA patients as compared to MI-CAD patients. The covariates used to assess the outcomes differed across the studies. The follow-up period in the studies ranged from 1 to 19.9 years. All studies were of good quality, with an NOS score of 8.

**Table 2:** Details of included studies

Study	Location	Groups	Sample size	Age (years)	Male gender (%)	HT (%)	DM (%)	CKD (%)	Previous MI (%)	Previous CVA (%)	Medications at discharge				Factors adjusted in multivariable analysis	Follow-up	NOS score
											ASA	P2Y12 inhibitor	Statins	Beta- blockers			
Lawless 2023 [17]	UK	MINOCA MI-CAD	1439 11,763	67 72	69.8 55.2	39.2 59.3	14.3 24.1	2.7 0.9	14.6 26.1	NR	NR	NR	NR	NR	Age, family history of coronary artery disease, hypercholesterolaemia, HT, smoking status, cardiogenic shock at the time of angiogram, and ST-segment elevation on baseline ECG	4.6 years	8
Magnani 2022 [24]	Italy	MINOCA MI-CAD	317 1683	38 41	75.7 91.3	18.3 28.3	3.8 8.4	NR	NR	NR	82 95.1	28.4 54.6	87.4 99.4	65.6 85.4	Age, sex, BMI, DM, HT, smoking habits, dyslipidemia, a family history of cardiac disease, previous thromboembolic events, hypertension, LVEF, medical treatment, and admission presentation	19.9 years	8
Vranken 2020 [13]	Netherlands	MINOCA MI-CAD	402 4025	64 68	48.5 74	48.6 58.2	13.8 18.5	5.2 4.4	5.8 15.7	2.8 3.9	61.8 87.4	29.2 74.1	NR	NR	Age, DM, current smoking, creatinine levels at admission	1 year	8
Lopez-Pais 2020 [23]	Spain	MINOCA MI-CAD	109 412	64.6 66.7	48.6 78.2	61.5 62.1	23.9 35.6	NR	NR	NR	NR	NR	NR	NR	Age, HT, dyslipidemia, DM, tobacco use	1 year	8
Gasior 2020 [22]	Poland	MINOCA MI-CAD	6063 160,886	67 65	46.9 65.6	73.9 73.3	22.4 25.8	NR	0 18	3.2 3.5	87.6 92.4	67.5 84.8	83.3 88.9	78.6 84.4	Age, gender, obesity, dyslipidemia, DM, smoking, pulmonary disease, peripheral artery disease, heart rate, chest pain, sinus rhythm, LVEF	1 year	8
Dreyer 2020 [21]	USA	MINOCA MI-CAD	16,849 269,931	75.1 75.6	23 58.5	NR	25.8 37.9	7.7 10.8	7.8 16.9	NR	NR	NR	NR	NR	Sociodemographics, cardiovascular history and comorbidities	1 year	8
Schmitz 2020 [20]	USA	MINOCA MI-CAD	73 2097	61.6 63.4	62.6 70.5	51.4 59.3	19.2 21.9	NR	4.1 15.5	2.7 5.3	NR	NR	NR	NR	Age, gender, history of heart failure, history of CABG, HT, hyperlipidemia, prior MI, prior percutaneous coronary intervention, current tobacco, current rural status, presentation with heart failure	1 year	8
Abdu 2019 [18]	China	MINOCA MI-CAD	128 1901	61.9 65.4	53.1 77.2	52.3 62.4	10.9 29.6	NR	2.3 10.9	13.3 15.4	83.6 94.8	48.4 92.5	87.5 95.6	52.3 72.6	Age, gender, HT, DM, smoking, atrial fibrillation, heart failure, cerebral infarction, total cholesterol, cardiac troponin and LVEF	1 year	8
Choo 2019 [19]	South Korea	MINOCA MI-CAD	396 10,871	62.3 63.4	57.3 74.9	50.8 48.9	22 26.5	NR	NR	5.8 6.2	95.5 99.7	36.9 96.5	73 91.9	33.6 83.5	Age, gender, Killip class at initial presentation, DM, current smoking, ST changes in the initial ECG, lipid profile, and LVEF	2 years	8
Williams 2018 [31]	New Zealand	MINOCA MI-CAD	897 7408	63.1 65.1	45.7 72.5	NR	15.7 22.7	NR	NR	NR	90.4 97.2	67.9 84.4	89.8 95.8	72.2 87.2	Age, gender, heart rate, systolic blood pressure, Killip score, cardiac arrest, ST-segment depression, elevated cardiac enzymes, creatinine. aspirin, statin, second antiplatelet agent, ACE inhibitor/angiotensin receptor blocker, beta-blocker	2 years	8
Raparelli 2018 [30]	Canada	MINOCA MI-CAD	82 916	49 49	58.5 67	32.9 36.9	7.3 16.9	NR	15.8 20.3	NR	85.4 98.4	NR	81.7 94.8	67.1 87.8	Age, gender, GRACE score, HT, PCS at baseline, therapy at discharge	1 year	8
Barr 2018 [29]	New Zealand	MINOCA MI-CAD	302 1768	56.9 60.7	50 74.4	NR	19.5 27	NR	NR	NR	97.4 99.4	68.9 78.8	93.4 97.4	77.2 89	Age, gender, heart rate, systolic blood pressure, Killip score, cardiac arrest, ST depression, elevated cardiac enzymes, creatinine	2.4 years	8
Bainey 2018 [27]	Canada	MINOCA MI-CAD	2092 33,836	59 63.3	47 74.3	NR	14.1 25.4	3.8 4.7	5 11.2	NR	NR	NR	NR	NR	Age, gender, time to catheterization from index hospitalization, MI type and Charlson Comorbidity Index	1 years	8
Andersson 2018 [26]	Denmark	MINOCA MI-CAD	298 4239	65 63	61 74	44 40	12 13	NR	12 13	7 5	NR	NR	NR	NR	Age, gender, body mass index, smoking, HT, hyperlipidaemia, DM, previous MI, previous stroke, and family history of CAD, and left bundle branch block	2.6 years	8
Planer 2014 [28]	USA	MINOCA MI-CAD	197 2245	54 60	46.7 68	52.6 58.4	18.4 26.6	NR	10.9 21.8	NR	81.9 87.5	NR	NR	NR	Age, gender, DM, HT, current smoking, hyperlipidemia, history of MI, renal insufficiency, ST-segment deviation ≥ 1 mm, and troponin level	1 year	8
Cortell 2009 [25]	Spain	MINOCA MI-CAD	64 440	60 66	42.2 73.9	51.6 60	20.3 38	NR	3.1 14.8	3.1 6.8	NR	NR	NR	NR	Age, DM, prior MI, renal failure, heart failure on admission	3 years	8

*MINOCA* Myocardial infarction with non- obstructive coronary arteries, *MI-CAD* Myocardial infarction with obstructive coronary artery disease, *LVEF* Left ventricular ejection fraction, *HT* Hypertension, *DM* Diabetes mellitus, *MI* Myocardial infarction, *CVA* Cerebrovascular accident, *CAD* Coronary artery disease, *CKD* Chronic kidney disease, *CABG* Coronary artery bypass grafting, *GRACE* Global Registry of Acute Coronary Events, *ACE* Angiotensin converting enzyme, *PCS* Physical component summary, *NR* Not reported, *NOS* New-castle Ottawa scale

### Outcomes

**Fig. 2:**
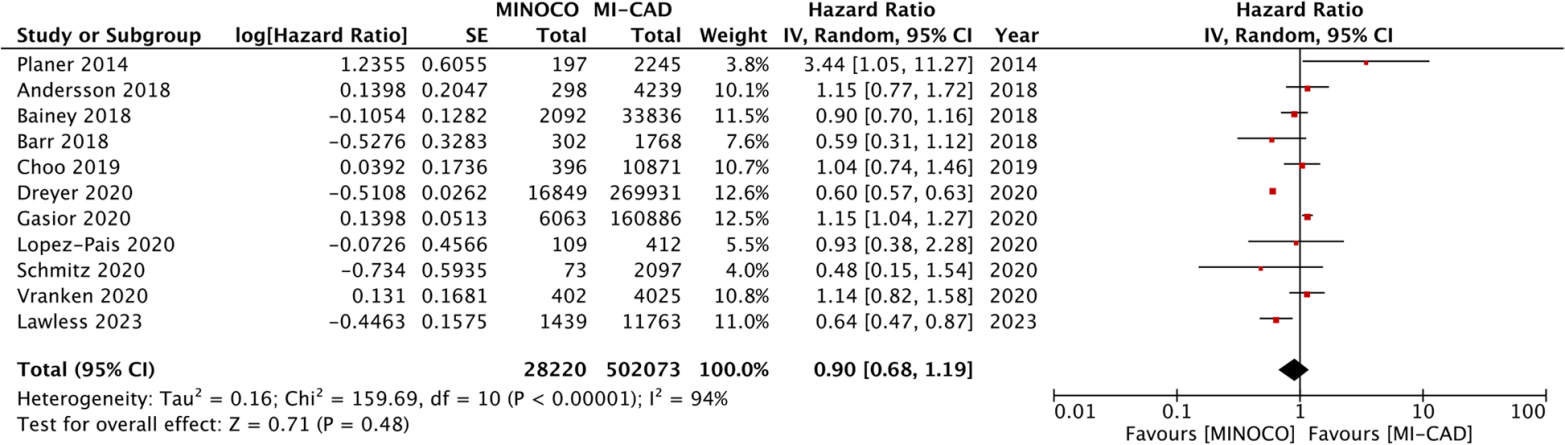
Meta-analysis of adjusted outcomes comparing all-cause mortality between MINOCA and MI-CAD

**Fig. 3:**
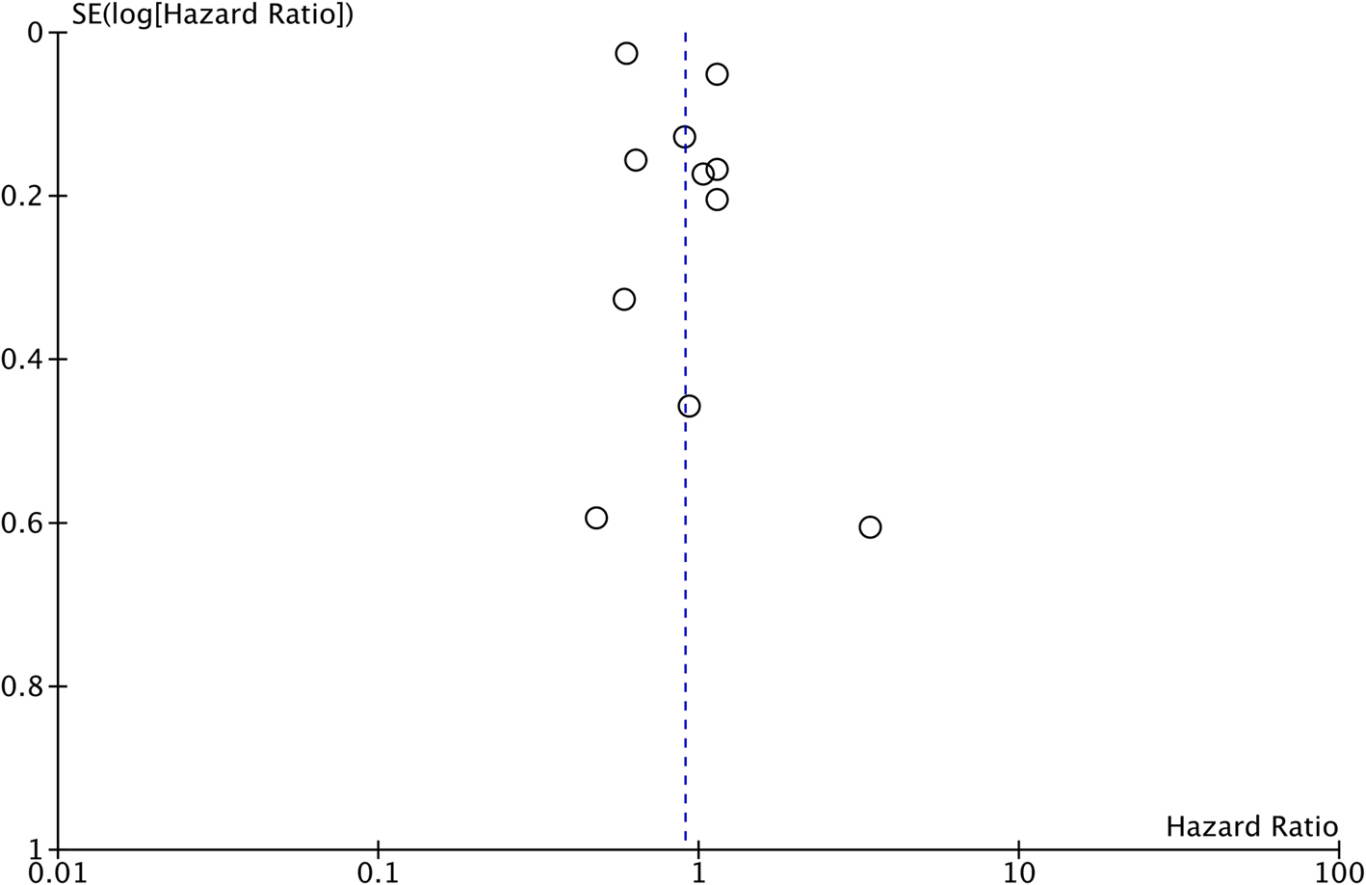
Funnel plot for the meta-analysis of all-cause mortality

A total of 28,220 patients with MINOCA were compared with 502,073 patients with MI-CAD in 11 studies reporting all-cause mortality. Our meta-analysis revealed no statistically significant difference in the risk of all-cause mortality between patients with MINOCA and MI-CAD (HR: 0.90 95% CI 0.68, 1.19 I^2^ = 94% *p* = 0.48) (Fig. 2). We did not find any gross asymmetry in the funnel plot (Fig. 3). Egger’s test did not indicate any publication bias (*p* = 0.77). Sensitivity analysis is shown in Table 3. There was no change in the significance of the outcome on the removal of any study and the overall results of all-cause mortality remained non-significant throughout. Data on cardiovascular mortality was reported only by three studies. Meta-analysis showed no statistically significant difference in the risk of cardiovascular mortality between MINOCA and MI-CAD cohorts (HR: 0.81 95% CI 0.54, 1.22 I^2^ = 0% *p* = 0.31) (Supplementary Fig. 1).

**Table 3:** Results of sensitivity analysis for all-cause mortality between MINOCA vs MI-CAD

Excluded study	Resultant effect size (Hazard ratios)
Planer 2014 [24]	0.89 (95% CI 0.65, 1.13 I^2^ = 94% *p* = 0.28)
Andersson 2018 [22]	0.88 (95% CI 0.66, 1.18 I^2^ = 94% *p* = 0.39)
Bainey 2018 [23]	0.91 (95% CI 0.67, 1.23 I^2^ = 94% *p *= 0.52)
Barr 2018 [25]	0.94 (95% CI 0.70, 1.25 I^2^ = 94% *p* = 0.66)
Choo 2019 [29]	0.89 (95% CI 0.60, 1.20 I^2^ = 94% *p* = 0.44)
Dreyer 2020 [19]	0.96 (95% CI 0.80, 1.77 I^2^ = 62% *p* = 0.71)
Gasior 2020 [20]	0.86 (95% CI 0.67, 1.11 I^2^ = 82% *p* = 0.26)
Lopez-Pais 2020 [21]	0.90 (95% CI 0.68, 1.20 I^2^ = 94% *p *= 0.48)
Schmitz 2020 [18]	0.93 (95% CI 0.70, 1.20 I^2^ = 94% *p* = 0.61)
Vranken 2020 [13]	0.88 (95% CI 0.65, 1.18 I^2^ = 94% *p* = 0.39)
Lawless 2023 [17]	0.94 (95% CI 0.70, 1.28 I^2^ = 94% *p* = 0.71)

*CI* Confidence intervals

**Fig. 4:**
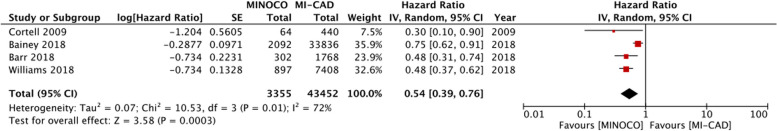
Meta-analysis of adjusted outcomes comparing all-cause mortality and MI between MINOCA and MI-CAD

**Fig. 5:**
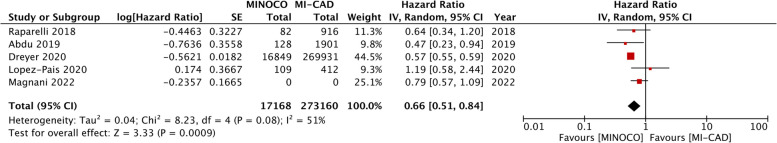
Meta-analysis of adjusted outcomes comparing MACE events between MINOCA and MI-CAD

**Fig. 6:**
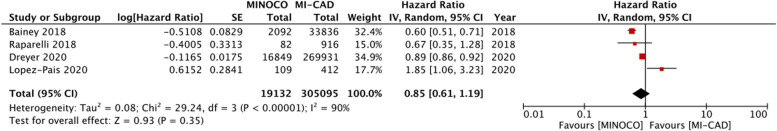
Meta-analysis of adjusted outcomes comparing readmission rates between MINOCA and MI-CAD

Four studies were reporting combined risk of death and MI between the two study groups. On pooled analysis of these studies, there was a statistically significant reduced risk of mortality and MI in patients with MINOCA vs MI-CAD (HR: 0.54 95% CI 0.39, 0.76 I^2^ = 72% *p* = 0.003) (Fig. 4). Similarly, analysis of five studies showed a significantly reduced risk of MACE in patients with MINOCA as compared to MI-CAD (HR: 0.66 95% CI 0.51, 0.84 I^2^ = 51% *p* = 0.0009) (Fig. 5). Lastly, a meta-analysis of data indicated no statistically significant difference in the risk of readmission between the two study groups (HR: 0.85 95% CI 0.61, 1.19 I^2^ = 90% *p* = 0.35) (Fig. 6).

## Discussion

Due to the widespread prevalence of coronary artery disease, several studies have focussed on assessing long-term outcomes and prognostic factors of MI in the past few years [32–34]. Indeed, MI is a well-defined life-threatening disease and the outcomes can differ due to several factors like patient’s age, gender, the severity of disease, presence of risk factors, co-morbidities, and treatment protocol [32]. Thus, assessment of long-term outcomes with any type of MI should also consider the parallel influence of these confounders to present correct scientific evidence. For instance, many studies have explored the impact of gender on outcomes of MI but with variable results based on crude or adjusted data [33, 35]. Bavishi et al. [35] in a comprehensive review have shown that while crude long-term mortality rates may be higher in females as compared to males[Risk ratio (RR) 1.60, 95% CI: 1.46–1.76], the risk is no longer statistically significant when adjusted effect estimates were pooled for a meta-analysis (RR: 1.01, 95% CI: 0.93–1.11). They concluded that baseline clinical differences and different treatment protocols largely contributed to the high crude mortality rates in female patients.

In this study, we attempted to extrapolate the same theory in assessing the long-term outcomes of patients with MINOCA compared to MI-CAD patients. Many of the studies comparing MINOCA and MI-CAD, reported a favourable prognosis in patients with MINOCA [9–11]. Bossard et al. [9] compared data of 1599 MINOCA patients with 22,184 MI-CAD patients, and have demonstrated significantly lower all-cause mortality, cardiovascular mortality, repeat MI and major bleeding episodes in MINOCA patients. Eggers et al. [36] in a retrospective analysis of a Swedish registry have shown lower rates of all-cause mortality, cardiovascular mortality, and MACE events in patients with MINOCA as compared to MI-CAD. Other studies from Germany [37] and Egypt [38] have also demonstrated more favourable outcomes in patients with MINOCA. In the prior review on this topic, Pelliccia et al. [14] have reported annual long-term mortality rates of 2.2% in patients with MINOCA and 5% in patients with MI-CAD. By compiling evidence from 26 studies, the authors reported a statistically significant 40% lower risk of all-cause mortality in patients with MINOCA as compared to MI-CAD (RR 0.60, 95% CI: 0.46 to 0.78).

While Pelliccia et al. [14] pooled only crude data, our review synthesized data of only adjusted effect estimates and presents contrasting results. Our analysis shows that long-term mortality does not significantly differ between the two disease types after adjusting for confounding factors. We acknowledge that the statistical power of our analysis would be lower as compared to the previous review as only 11 studies were available in our primary analysis despite extending the literature search by five more years and adding recent studies. However, the sample size of our analysis was large, with data of 28,220 patients with MINOCA and 502,073 patients with MI-CAD. Furthermore, sensitivity analysis demonstrated that no study in our analysis had a disproportionate impact on the overall outcome. Forest plot analysis showed that the study of Dreyer et al. [21], with its significantly large sample size, may be considered an outliner. The authors of this study noted a significant higher risk of all-cause mortality in MINOCA after adjusting for past cardiovascular history and comorbidities. These difference in their results as compared to other studies may be attributable to two reasons. First, a study by Dreyer et al. only included elderly patients (≥ 65 years). Secondly, over 50% of patients were eventually excluded due to incompleteness or lack of data linkage. This suggests possible selection bias, and may impact the generalizability of the results.

The outcomes of our study should be interpreted while considering the differences in the factors adjusted in the included studies. The most common adjusted confounders were age, gender, and comorbidities like diabetes, hypertension, and dyslipidaemia. Several studies have demonstrated that patients with MINOCA are younger and more often of female gender [4, 12]. Consistent with younger age, these patients may also have a lower prevalence of other risk factors such as diabetes, smoking, hypertension, renal disease, history of MI, and stroke [39]. However, a systematic review had indicated that cardiovascular risk factors are not different in MINOCA and MI-CAD patients [4]. This could explain the lack of difference in all-cause mortality between MINOCA and MI-CAD in the current meta-analysis. Moreover, unlike MI-CAD, no clear management strategy exist for MINOCA, and it differs from case to case. Similar to heart failure, MINOCA is considered a working diagnosis that requires further evaluation to identify the underlying cause. Further investigations like transthoracic echocardiography and magnetic resonance imaging are needed to tailor the treatment based on the underlying pathology [3]. Research also indicates that secondary prevention strategies are less commonly utilized in MINOCA as compared to MI-CAD. Renin-angiotensin inhibitors may have a beneficial role but dual antiplatelet therapy and statins offer no advantage in MINOCA patients [40]. Thus, it is evident that the outcomes of these conditions may be influenced only by the differences in the baseline characteristics but also by the variability in the management protocols. The lack of clear management strategy and lower utilization of prevention protocols could be another reason for similar mortality of MINOCA and MI-CAD despite the younger age of the MINOCA group.

In our secondary analysis, we noted a significantly reduced risk of combined mortality and MI as well as MACE in patients with MINOCA as compared to MI-CAD. These results should be interpreted with extreme caution due to limited data and small sample size of the studies. There is a need for further research to explore the differences in the risk of cardiovascular mortality, MACE and readmission rates between MINOCA and MI-CAD patients.

Our review has limitations. Firstly, only eleven studies provided data on long-term all-cause mortality. We had to exclude many studies due to the lack of adjusted data. Thus, our analysis does not encompass the entirety of evidence available in the literature. Secondly, we noted high heterogeneity in the meta-analysis which partly could be due to differences among the included studies for the factors adjusted in the multivariable analysis. It is possible that other measured and unmeasured factors in the included studies could have affected the outcomes. Thirdly, all included studies were retrospective with inherent bias associated with these types of studies. Fourthly, we could include maximum studies only in the primary outcome analysis. We were unable to comprehensively analyse other important outcomes like cardiovascular mortality, recurrent MI, MACE, and readmission rates due to limited data. Lastly, the software RevMan used in our meta-analysis uses the DerSimonian & Laird Method to calculate error rates and can result in false positive results with scarce data.

A major strength of our study is that this is the first meta-analysis comparing mortality rates between MINOCA and MI-CAD by pooling adjusted data. The consistency of the outcomes on leave-one-out analysis lends credibility to our conclusions. The contrasting results presented by our study as compared to the previous review [14] have important clinical implications as they suggest that MINOCA should not be considered a benign entity as compared to MI-CAD. Clinicians should aggressively search for the underlying pathology to adequately manage this disease. Further research should be conducted to identify specific risk factors associated with poor outcomes with MINOCA.

To conclude, this is a large meta-analysis of 16 studies, reporting only adjusted and long-term differences between MINOCA and MI-CAD patients. We show that there is no difference in the risk of all-cause mortality between the two types of disease. The consistency of the results on sensitivity analysis indicated robustness of our evidence. No difference between MINOCA and MI-CAD was detected in terms of the cardiovascular mortality. Limited evidence also showed reduced risk of MACE in MINOCA vs MI-CAD but no difference in the risk of readmissions between the two conditions. Our results should be interpreted cautiously due to the high heterogeneity in the meta-analysis and limited data on cardiovascular mortality, MACE, and readmissions. Further research is needed to strengthen the evidence on this topic.

### Supplementary Information

**Additional file 1: Supplementary figure 1.**

**Additional file 2. **Supplementary material: Raw data.

## Data Availability

All data generated or analysed during this study are included in this published article and its supplementary information.
