# Supplementary figures and images for: Long term all-cause mortality after myocardial infarction with non-obstructed vs obstructed coronary artery disease: a meta-analysis of adjusted data
Source: BMC Cardiovasc Disord. 2024 Jan 2;24:9. doi: 10.1186/s12872-023-03674-1 (PMC10763149; doi:10.1186/s12872-023-03674-1)

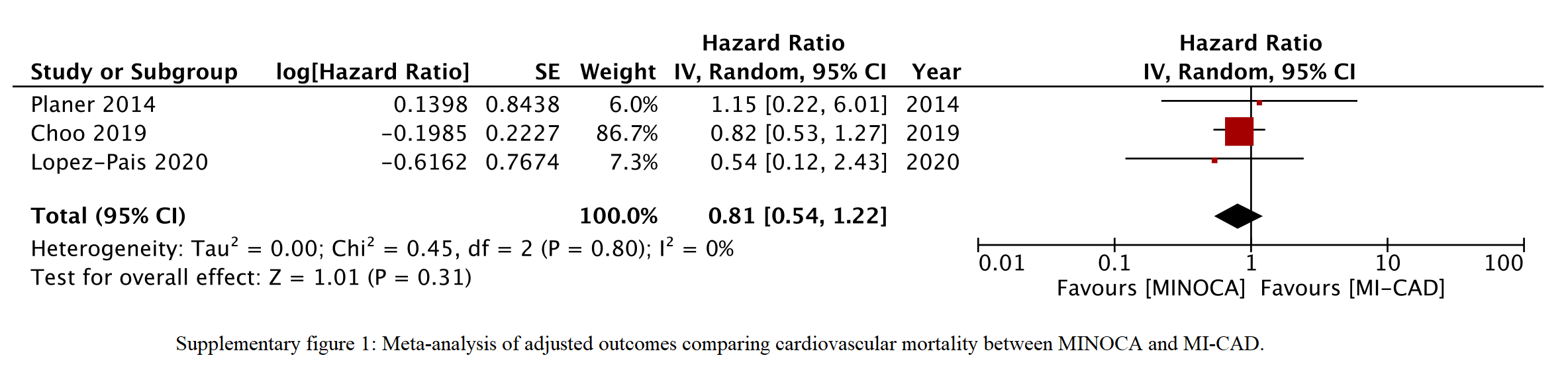

Supplement: Supplementary file 1 — Additional file 1: Supplementary figure 1. [file 12872_2023_3674_MOESM1_ESM.tiff]
